# Supplementary material for: The intracellular seven amino acid motif EEGEVFL is required for matriptase vesicle sorting and translocation to the basolateral plasma membrane
Source: PLoS One. 2020 Feb 12;15(2):e0228874. doi: 10.1371/journal.pone.0228874 (PMC7015431; doi:10.1371/journal.pone.0228874)
Supplement: Attachment — Submitted filename: PLOS ONE D-19-15523.pdf [file pone.0228874.s005.pdf]

The aim of the study was to identify and characterize a cytoplasmic basolateral sorting signal in the transmembrane serine protease, matriptase. In fact, a basolateral sorting motif in the protein, which resembles the sequence responsible for basolateral sorting of the polymeric immunoglobulin receptor, has been identified in previous studies. However, the authors have decided to revisit the question since that signal does not contain canonical tyrosines or leucines, and is not conserved among species. Since the enzyme is an important one, and since polarized sorting could play an important role in its function in health and disease, the research aim is important and relevant.

Using site-directed mutagenesis and cell biological (mainly morphological)-based assays, the authors propose that a basolateral sorting motif that is based on leucine and an acidic cluster exists in the cytoplasmic domain of matriptase. Notably, all research was performed on a matriptase N-terminal fragment ending at amino acid Gly-149 fused at its extracellular C-terminus to eGFP.

Critiques:

I feel that the reported data do not firmly imply that the identified signal is indeed a basolateral sorting signal. Therefore, a significant amount of research has to be invested to address this point properly.

1). The importance of Figs 1 & 2 is to show that the ectopically expressed fusion protein localization is approximately normal. This was done by comparing its localization with respect to the endogenous protein (stained with the M24 antibody that recognizes the endogenous, but not the ectopically expressed protein) in Fig. 1, and by showing the preferential plasma membrane localization of the expressed protein with respect to ZO-1 staining, in Fig. 2. I think that the data in Fig.2 are not important for the studied question. In addition, deletion of cytoplasmic tails of transmembrane proteins typically have gross and often times artifactual effects on membrane trafficking of the mutated protein.

2). Regarding data presented in Fig. 4. The resolution of this image (as well as of other images) was typically low, posing a difficulty to understand the details that the authors have pointed out in the text. For instance, I did not get their interpretation that the regions marked with arrows and appear in green mark the cell periphery. The different mutants presented show ER, or possibly some Golgi-like staining. In fact, the subtitle given to the related Figure 6 may support this idea. But, since there was not an attempt to address this point, it is hard to say where these mutants are located within the cells. I would also suggest adding the MTPN-wt distribution to the composite to facilitate the comparison. Nonetheless, this is an important point because if indeed the protein is stuck in the early secretory pathway, then the basolateral sorting signal is not at all such a signal, but an ER or Golgi localization signal. Typically, inactivation of a basolateral sorting signal causes the mislocalization of a protein to the apical surface and has no significant impact on the exit of the mutated protein from the ER or Golgi.

3). As per the experiments described in Fig. 5, which assessed the effects of the mutated residues on the apical and basal localization of the protein in polarized MDCK cells. To verify that the mutated versions of the protein are indeed mislocated to the apical surface, the authors have to perform genuine surface stainings of the protein, using at least one of the two indicated approaches; a) performance of surface labeling of the protein using antibodies directed against the ectodomain of the protein, and under conditions whereby the cells are not permeabilized. In this strategy, providing confocal x-z images is not sufficient, and quantitative analysis of the Ap vs. Bl distribution of the protein should be provided. b) performance of cell-surface biotinylation/biochemical based assay in which cell surface proteins are biotinylated and pulled down and the protein of interest is detected by Western blotting. These experiments are fundamental for the proper addressing the effects of the mutations on the polarized distribution of matriptase.

4). A prominent hallmark of basolateral sorting signals is that they can act in a dominant and autonomous manner. Thus, it would be essential to show that the signal identified in this study can confer basolateral sorting of a heterologous protein that is intrinsically sorted to the apical surface, and that the mutations abrogate this process.

5). I found the paper difficult to read, containing unexplained jargon and abbreviations, s as well as long and unclear sentences (e.g., in page 11 the sentence starting with " The lack of significant…", or "The targeting of MTPN-EGFP to cell periphery…").
