# Supplementary material for: The intracellular seven amino acid motif EEGEVFL is required for matriptase vesicle sorting and translocation to the basolateral plasma membrane
Source: PLoS One. 2020 Feb 12;15(2):e0228874. doi: 10.1371/journal.pone.0228874 (PMC7015431; doi:10.1371/journal.pone.0228874)
Supplement: Attachment — Submitted filename: Rebuttal letter 3.docx [file pone.0228874.s007.docx]

Dear Dr. Sang,

Thank you for your consideration of our paper and the thoughtful previous and current comments of reviewer#1. We are, however, somewhat disappointed that Reviewer #2’s current comments are very different from their previous review and appear to indicate lack of understanding of the field, and unfortunately misrepresentation of our data. Given this, we wonder if Reviewer #2 might have had a junior member of their group conduct the review? We have addressed the specific comments below:

Reviewer #1:

Critiques:
Reviewer #1: The authors should have stated the "not sufficient" statement in the manuscript and not only in the rebuttal letter. For instance, in the Abstract it should have been stated: " Our study reveals that (the word "that" was missing there) the EEGEVFL motif is necessary, but may not be sufficient, for matriptase basolateral membrane targeting....".

**Response:** We agree with Reviewer #1 that although our data only support a required role of the seven amino acid motif in matriptase basolateral targeting. We have added the statement of “but may not be sufficient” in the abstract.

Reviewer #2:

This is a revised manuscript. However, some points in the revised manuscript are not well addressed.

1. The real colors of the fluorescence images (EGFP, M24 and ZO-1) in Figure 2, 3, 4 and 5 were not individually shown in the revised manuscript. The image qualities in the revised manuscript are not improved.

**Response:** We do not understand what Reviewer #2 means by “real colors”. When a digital imaging system is used as herein, the images are acquired in a numerical format as intensity per pixel. Thus, the most typical representation of the data converts intensity into grayscale. “False colors” can then assigned to the black-and-white digital images by computer software. For data presentation, grayscale images give the best representation of contrast and details. When colocalizations studies are conducted for more than one species, false color representations are useful for comparisons. We respectfully contend that the individual images with false color applied will not provide better quality than the ones in black-and-white.

2. In Figure 4B, some of the EGFP images were extended out of the ZO-1-stained borders. What reasons cause the phenomena? Whether those matriptase-EGFP fusion proteins will affect the formation of tight junctions?

**Response:** It is not uncommon to observe basolateral membrane ruffles in polarized cells. The ZO-1 staining defines the tight junction, however, it is not confined to the border of the cell. It has been documented that the basolateral “body” can be larger than the area defined by the tight junctions. <https://www.researchgate.net/figure/Z-stack-image-analysis-of-representative-bestrophin-1-mutants-in-MDCK-II-cells-A_fig4_51606308>

In addition, in Figure 5, ZO-1 was detected at the tight junction and we came to the conclusion that matriptase-EGFP does not alter tight junction formation.

3. Matriptase has been shown to be mainly located in the adherin junction region of polarized MDCK cells. However, MTPN-EGFP proteins looked like to be mainly co-localized with ZO-1 at the apical surface in Figure 3 and 5 of the current manuscript. It raises a question whether MTPN-EGFP can behave like a real matriptase for the analysis of the protease transcytosis. The authors should explain why MTPN-EGFP proteins did not well exhibit the real localization of matriptase in the differentiated epithelial cells?

**Response:**

ZO-1 is a well-established **tight junction** marker, which was used to show the coincidence of matriptase with ZO-1 on the surface of MDCK cells in Figure 3. Our statement regarding this observation is “*The peripheral staining was further confirmed by its coincidence with ZO-1, a well-established tight junction marker (Fig. 3 A, B and C, as indicated by arrowheads)*.” We carefully used “coincidence” rather than “co-localization” to avoid some confusions. We never stated that “*MTPN-EGFP proteins looked like to be mainly co-localized with ZO-1 at the apical surface in Figure 3 and 5 of the current manuscript.*” Tight junctions are not on the apical surface. It is factually incorrect for Reviewer #2 to say that ZO-1 and matriptase are colocalized on the apical surface. TMPRSS2 is on the apical surface.

The comment regarding Figure 5 further demonstrate Reviewer #2’s confusion. Should two signal occupy the same position in the XY plane, but be located at a different depth, a sample imaged at the XY orientation may show the two signals colocalizing, depending on the depth of the field (as seen in Figure 3). However, if a sample is imaged and represented in the ZY, or the ZX orientation, it would reveal that the two signals do not in fact colocalize (as seen in Figure 5). Our data showed MTPN-EGFP coincides with ZO-1 in Figure 3, because it is a micrograph imaged at the XY orientation. To further dissect the subcellular location of MTPN-EGFP, we imaged the cells at the XZ orientation, thus revealing that MTPN-EGFP is located at the basolateral surface. Our data characterizes matriptase localization at a higher resolution.

In a previous study [1], we demonstrated that endogenous matriptase was detected at the adherens junctions in 184 A1N4 human mammary epithelial cells. In another previous study [2], we showed that endogenous matriptase was detected on the basolateral plasma membrane in differentiated Caco-2 human colon adenocarcinoma cells. Our matriptase mAbs do not cross-react with canine matriptase, and to the best of our knowledge, there have been no reports that commercially available matriptase antibodies can detect canine matriptase. We, therefore, believe that Reviewer #2 must simply be mistaken in their assertion that matriptase has been shown to be mainly located in the adherins junctions of polarized MDCK cells, as we are unaware of any such study, published or presented in a scientific meeting. If by “real” matriptase, Reviewer #2 means “endogenous” matriptase in Caco-2 cells rather than in MDCK cells, the data and conclusions of our current study using MDCK cells with an engineered matriptase fusion protein are entirely consistent with our published data studying endogenous matriptase in Caco-2 cells [2]. The coincidence of endogenous matriptase and ZO-1 in the XY orientation and the differential localization of endogenous matriptase in the basolateral surface versus ZO-1 in the tight junction in ZY orientation in Caco-2 human colon adenocarcinoma cells can be found in our previous study, which is also presented here .

Based on these data we believe that the engineered model system we have used in this study replicates the behavior of “real” matriptase with great fidelity.

References

1.	Hung RJ, Hsu I, Dreiling JL, Lee MJ, Williams CA, Oberst MD, Dickson RB, Lin CY (2004) Assembly of adherens junctions is required for sphingosine 1-phosphate-induced matriptase accumulation and activation at mammary epithelial cell-cell contacts. Am J Physiol Cell Physiol 286: C1159-C1169.

2.	Wang JK, Lee MS, Tseng IC, Chou FP, Chen YW, Fulton A, Lee HS, Chen CJ, Johnson MD, Lin CY (2009) Polarized epithelial cells secrete matriptase as a consequence of zymogen activation and HAI-1-mediated inhibition. Am J Physiol Cell Physiol 297: C459-C470.
